# Characterization of gut microbiota profiles by disease activity in patients with Crohn’s disease using data mining analysis of terminal restriction fragment length polymorphisms

**DOI:** 10.3892/br.2014.252

**Published:** 2014-03-14

**Authors:** AKIRA ANDOH, TOSHIO KOBAYASHI, HIROYUKI KUZUOKA, TOMOYUKI TSUJIKAWA, YASUO SUZUKI, FUMIHITO HIRAI, TOSHIYUKI MATSUI, SHIRO NAKAMURA, TAKAYUKI MATSUMOTO, YOSHIHIDE FUJIYAMA

**Affiliations:** 1 Division of Mucosal Immunology, Graduate School of Medicine, Shiga University of Medical Science, Otsu, Shiga 520-2192, Japan; 2 Miyagi University, Sendai, Miyagi 982-0215, Japan; 3 Research and Development Laboratories, EN Otsuka Pharmaceutical Co., Ltd., Hanamaki, Iwate 025-0312, Japan; 4 Department of Medicine, Shiga University of Medical Science, Otsu, Shiga 520-2192, Japan; 5 Department of Internal Medicine, Sakura Medical Center, Toho University, Sakura, Chiba 285-8741, Japan; 6 Department of Gastroenterology, Chikushi Hospital, Fukuoka University, Chikushino, Fukuoka 814-0180, Japan; 7 Division of Lower Gastroenterology, Department of Internal Medicine, Hyogo College of Medicine, Nishinomiya, Hyogo 663-8131, Japan

**Keywords:** data mining, microbiota, terminal restriction fragment length polymorphism, inflammatory bowel disease

## Abstract

The gut microbiota plays a significant role in the pathogenesis of Crohn’s disease (CD). In this study, we analyzed the disease activity and associated fecal microbiota profiles in 160 CD patients and 121 healthy individuals. Fecal samples from the CD patients were collected during three different clinical phases, the active (n=66), remission-achieved (n=51) and remission-maintained (n=43) phases. Terminal restriction fragment length polymorphism (T-RFLP) and data mining analysis using the Classification and Regression Tree (C&RT) approach were performed. Data mining provided a decision tree that clearly identified the various subject groups (nodes). The majority of the healthy individuals were divided into Node-5 and Node-8. Healthy subjects comprised 99% of Node-5 (91 of 92) and 84% of Node-8 (21 of 25 subjects). Node-3 was characterized by CD (136 of 160 CD subjects) and was divided into Node-6 and Node-7. Node-6 (n=103) was characterized by subjects in the active phase (n=48; 46%) and remission-achieved phase (n=39; 38%) and Node-7 was characterized by the remission-maintained phase (21 of 37 subjects; 57%). Finally, Node-6 was divided into Node-9 and Node-10. Node-9 (n=78) was characterized by subjects in the active phase (n=43; 55%) and Node-10 (n=25) was characterized by subjects in the remission-maintained phase (n=16; 64%). Differences in the gut microbiota associated with disease activity of CD patients were identified. Thus, data mining analysis appears to be an ideal tool for the characterization of the gut microbiota in inflammatory bowel disease.

## Introduction

Inflammatory bowel disease (IBD), comprising ulcerative colitis (UC) and Crohn’s disease (CD), is a chronic intestinal disorder of unknown etiology (1–4). The pathogenesis of IBD involves an aberrant response by the mucosal immune system toward luminal antigens, such as dietary factors and/or commensal microbiota in genetically susceptible individuals (2,5–8). In particular, the commensal microbiota is regarded as the major environmental factor associated with IBD (5,7,9–12). IBD is mainly localized to those intestinal areas in which the majority of the bacteria are congregated, namely, the distal small intestine and the colon. The commensal microbiota is essential for the development of experimental colitis in various animal models of IBD (6,9).

The global composition of the gut microbiota, rather than the presence of certain pathogens, is most relevant to the etiology and pathogenesis of IBD (dysbiosis hypothesis) (5,13–16). Molecular approaches targeting the 16S ribosomal (r)DNA have been used to define significant changes in the diversity and composition of the gut microbiota in IBD (17). For example, a marked decrease in the relative abundance of members of the phylum Firmicutes, particularly *Clostridium* clusters IV and XIV, has been reported in IBD (5,17,18). The etiological significance of this finding is supported by a recent study by Atarashi *et al* (19), which demonstrated that the genus *Clostridium* plays a significant role in the induction of colonic regulatory T cells, which play a central role in maintaining immune homeostasis. Other reports indicated that *Faecalibacterium prausnitzii*, a member of *Clostridium* cluster IV, is also clinically significant (20–22).

It was previously demonstrated, using terminal restriction fragment length polymorphism (T-RFLP) analysis, that the fecal microbiota profile of CD patients differs from that of healthy individuals (14,18). This difference was observed even in patients with inactive disease (18). However, no differences associated with the activity of the disease were detected in the fecal microbiota profiles of CD patients with active and inactive disease. To further investigate the fecal microbiota of CD patients, we performed a data-mining analysis on the T-RFLP results from the analysis of fecal samples collected at three clinical time points (prior to induction therapy, immediately after the achievement of remission and ≥6 weeks later, while under continuous remission).

## Materials and methods

### Patients and samples

The patients and fecal samples used in this study were the same as those used in our previous study (14). In total, 66 patients with active CD [CD activity index (CDAI)>150 as reported by Best *et al* (23)] were recruited. The diagnosis of CD was based on clinical, endoscopic and pathological criteria. A total of 121 healthy individuals residing close to each center were also enrolled.

Fecal samples were collected from each patient at three different clinical phases: i) active disease at entry (active phase), ii) immediately after achievement of remission (CDAI<150; remission-achieved phase) and iii) maintained remission for ≥6 weeks (remission-maintained phase). The average period of remission between ii) and iii) was 15.7±10.8 weeks (mean ± SD). Samples from patients with ileostomy, patients who received surgical treatment or those who failed to achieve remission during the course of the study were excluded.

This study was approved by the Institutional Review Boards and the patients provided written informed consent prior to enrolment.

### DNA extraction

Each fecal sample (0.5 g) was suspended in 5 ml of Tris-EDTA buffer (pH 7.5) and centrifuged. This washing step was repeated 4 times. The sample was then resuspended in 5 ml of the same buffer containing lysozyme (5 mg/ml; Sigma, St. Louis, MO, USA), *N*-acetylmuramidase (0.5 mg/ml; Sigma) and achromopeptidase (0.5 mg/ml; Sigma). The following manipulations of DNA extraction were performed as previously described (24) and the final concentration of the DNA sample was adjusted to 20 ng/μl.

### Polymerase chain reaction (PCR) amplification and T-RFLP analysis

The 16S rRNA gene was amplified from human fecal DNA using the 27 forward 5′-AGAGTTTGATCCTGGCTCAG-3′ and 1492 reverse 5′-GGTTACCTTGTTACGACTT-3′ primers (25,26). The 5′-ends of the forward primers were labeled with 6′-carboxyfluorescein, which was synthesized by Applied Biosystems (Tokyo, Japan). The PCR amplifications of the DNA samples (10 ng of each DNA) were performed as previously described (25,26). The amplified 16S rDNA genes were purified using polyethylene glycol (PEG 6000) and redissolved in 20 μl distilled water.

The restriction enzymes were selected according to Matsumoto *et al* (25). The purified PCR products (2 μl) were digested with 20 U *Hha*I and *Msp*I at 55°C for 1 h. The length of the T-RF fragments was determined with an ABI PRISM^®^ 3100 or ABI 3130×l genetic analyzer (Applied Biosystems) in GeneScan mode. Standard size markers, such as GS500 ROX and GS1000 ROX (Applied Biosystems), were used. The fragment sizes were estimated using the local Southern method in GeneScan 3.1 software (Applied Biosystems). As the apparent size of identical T-RFs may vary by 1–2 bp among different gels and/or lanes of the same gel, major T-RFs similar in size by 1–2 bp were summarized to operational taxonomic units (OTUs). The major T-RFs were identified by computer simulation, which was performed using a T-RFLP analysis program (27), a phylogenetic assignment database for T-RFLP analysis of human colonic microbiota (25) and Microbiota Profiler (InfoCom T-RFLP Database & Analysis Software, Infocom Co., Tokyo, Japan). T-RFs with a peak height <25 fluorescence units were excluded from the analysis. Cluster analyses were performed using BioNumerics software (Applied Maths, Kortrijk, Belgium) based on the *Hha*I or *Msp*I T-RFLP patterns. The distances were calculated to determine any similarity among the samples and were graphically represented by constructing a dendrogram. Pearson’s similarity coefficient analysis and the unweighted pair-group methods with arithmetic means were used to establish the type of dendrogram.

### Data mining

Data mining analysis was performed using SPSS Clementine 14 software (IBM, Tokyo, Japan). A dividing system using the Classification and Regression Tree (C&RT) approach, which is the most typical method for constructing decision trees, using the Gini coefficient (28) between geographic districts and OTU data was applied. The records were divided into two subsets, so that the records within each subset were more homogeneous compared to the previous subset. C&RT is quite flexible and allows unequal misclassification costs to be considered, unlike other growing systems of data mining.

## Results and Discussion

**Figure 1:**
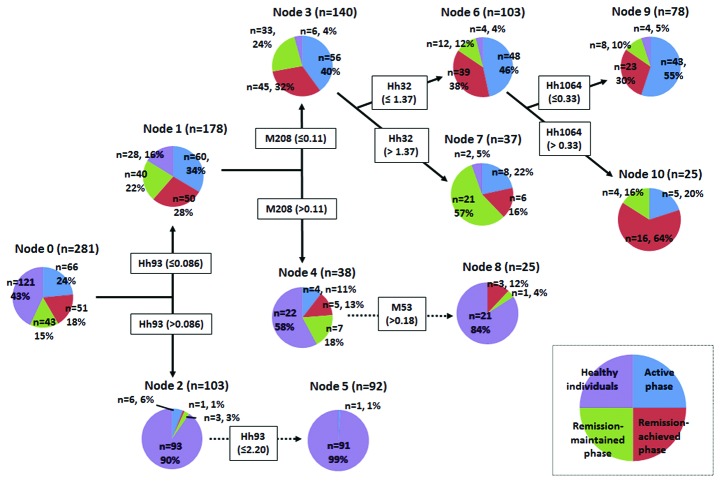
Decision tree constructed using the Classification and Regression Tree (C&RT) approach. Each operational taxonomic unit (OTU) is expressed as a restriction enzyme and RF length (bp), e.g. *Hha*I 93-bp OTU is abbreviated as Hh93 and *Msp*I 208-bp OTU is abbreviated as M208. The cut-off value of each dividing OTU was calculated from the OTU data of all the subjects, using the Gini coefficient with the C&RT method. Similar steps were repeated for the construction of a decision tree. Node-0 (the left end of the decision tree) is referred to as the root node, which is the starting point for tree construction. The details of the decision tree and the pathway indicate the species and quantities of OTUs, which contribute to dividing the various subject groups. RF, restriction fragment.

Data mining provided a decision tree as shown in Fig. 1, which clearly identified the various subject groups (nodes). A decision tree is a decision-supporting pathway that forms a tree-like graph. Each OTU was expressed as a restriction enzyme and RF length (bp), e.g., the *Hha*I 32-bp OTU was abbreviated as Hh32 and the *Msp*I 225-bp OTU was abbreviated as M225. Node-0 (the left end of the decision tree) is referred to as the root node, which is the starting point for tree construction and the decision tree grew toward the right to divide the subjects. As shown in Fig. 1, Node-0 was divided into Node-1 and Node-2 by Hh93, with a cut-off value of 0.086. This cut-off value was calculated from Hh93 data for all the subjects using the Gini coefficient and the C&RT method. Similar steps were repeated to fully construct the decision tree. The details of the decision tree and the pathway to the next node clearly indicated the species and quantities of OTUs, which contributed to the division of the various subject groups.

Node-1 included almost all the CD subjects and a small number of healthy subjects. By contrast, Node-2 consisted primarily of healthy subjects. These data indicate that Hh93 plays a significant role in the discrimination of healthy individuals from those with CD. Hh93 also played a role in the discrimination of the healthy individuals of Node-2 into Node-5. The database assignment of Hh93 included *Desulfovibrio* (a genus of sulfate-reducing bacteria) and *Lawsonia*; however, the pathological roles of these bacteria in human disease have not been clearly determined.

Node-1 was divided into Node-3 and Node-4 by M208, with a cut-off value of 0.11. Node-3 was characterized by CD patients in all phases; however, Node-4 consisted of 22 healthy individuals (58%) and 16 subjects with CD (42%), indicating that the gut microbiota profile of certain CD patients resembles that of healthy individuals. The database assignment of M208 included *Coprococcus*, *Roseburia*, *Dorea* and *Blautia*; however, the role of these bacteria has not been fully elucidated. M53 (*Faecalibacterium*), which had a cut-off value >0.18, led to further segregation of healthy individuals from Node-4 into Node-8.

As shown in Fig. 1, Node-3 included 56 CD subjects in the active, 45 in the remission-achieved and 33 in the remission-maintained phase. Node-3 was divided into Node-6 and Node-7 based on Hh32 (*Faecalibacterium*, *Bacteroides*), with a cut-off of 1.37. Node-6 included 48 subjects in the active phase (46%) and 39 subjects in the remission-achieved phase (38%), indicating that there are no significant differences in fecal microbiota profiles between CD patients in the active and remission-achieved phases. By contrast, Node-7 was characterized by 21 subjects in the remission-maintained phase (57%), indicating that the gut microbiota profile tends to change according to the duration of remission maintenance. Hh32 is assigned to *Faecalibacterium* and remission maintenance may stimulate the growth of this bacterium, which exhibits strong anti-inflammatory activity (20–22).

We previously reported the results of cluster analyses of the gut microbiota profiles of the same samples used in the present study (14). However, disease-associated differences were not identified, possibly due to the several limitations of the cluster analysis. For example, the cluster analysis only shows some classified groups and it does not produce clearly defined reasons for the creation of these groups. In addition, the obtained clusters lack flexibility, meaning that a slight modification of the data affects cluster formation. Furthermore, data mining constructs a decision tree, which is a set rule that predicts target variables and enables the creation of classification trees by repeated data division. During this process, a tree branch is formed and every branch determines the classification criteria for the dividing data. Therefore, exploration of a dataset by data mining enables the researcher to predict the most significant predictor variable. Additionally, once the decision tree is constructed, all the subsequent new records may be run with the same data mining tree, as long as the basic concepts of the data remain active. The main difference between data mining and cluster analysis is the capacity for handling data noise. Data mining skips characteristic noise and selects a series of related fields; however, cluster processing respects all data, without consideration of any numerical noise. Thus, in the present study it was possible to demonstrate geographical differences in the human gut microbiota in Japan.

In conclusion, to the best of our knowledge, this study is the first to identify disease activity and associated differences in the gut microbiota profiles of CD patients, which differ from those of healthy individuals. Among the CD patients, the gut microbiota profiles may differ according to disease activity. These results indicate that data mining is an ideal tool for characterizing human gut microbiota. Further investigations of the gut microbiota profiles associated with CD may lead to improved diagnostics and the development of novel therapeutic agents.
